# Emerging mechanisms of dynein transport in the cytoplasm versus the cilium

**DOI:** 10.1042/BST20170568

**Published:** 2018-07-31

**Authors:** Anthony J. Roberts

**Affiliations:** Institute of Structural and Molecular Biology, Birkbeck, University of London, Malet Street, London, U.K.

**Keywords:** cilia, dynein, intraflagellar transport, kinesin, microtubule

## Abstract

Two classes of dynein power long-distance cargo transport in different cellular contexts. Cytoplasmic dynein-1 is responsible for the majority of transport toward microtubule minus ends in the cell interior. Dynein-2, also known as intraflagellar transport dynein, moves cargoes along the axoneme of eukaryotic cilia and flagella. Both dyneins operate as large ATP-driven motor complexes, whose dysfunction is associated with a group of human disorders. But how similar are their mechanisms of action and regulation? To examine this question, this review focuses on recent advances in dynein-1 and -2 research, and probes to what extent the emerging principles of dynein-1 transport could apply to or differ from those of the less well-understood dynein-2 mechanoenzyme.

## Introduction

**Figure 1.**
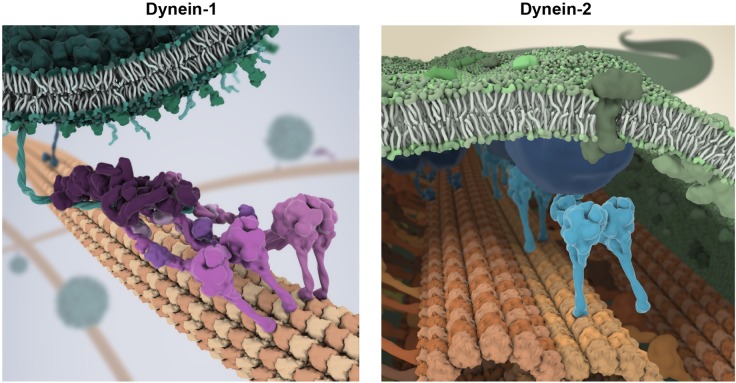
Speculative impressions of cargo transport by dynein-1 and dynein-2. Left: Depiction of dynein-1 transporting a vesicle. Two dynein-1 complexes (magenta) are templated by dynactin (dark purple) and a coiled-coil cargo adaptor (teal), whose distal end attaches to a receptor on the vesicle surface. For clarity, the densely packed milieu of molecules in the cytoplasm is not shown. Right: Depiction of dynein-2 (cyan) propeling a retrograde IFT train (blue) within the cilium. Dynein-2 operates in the confined space between the ciliary membrane (green) and the axoneme (orange), moving on the A-tubule of the microtubule doublet. Artwork in collaboration with Bara Krautz (www.scienceanimated.com; email: bara@scienceanimated.com).

Dyneins are minus-end-directed microtubule motor proteins in eukaryotic cells, built around force-generating subunits termed ‘heavy chains’. Based on phylogenetic analysis, the last eukaryotic common ancestor possessed a rich inventory of at least nine classes of dynein heavy chain [1,2]. In some eukaryotic lineages, these dynein classes expanded and diversified. For example, there are 16 dynein heavy chain genes in the human genome and 25 in the ciliate *Tetrahymena thermophila* [3,4]. The majority of these genes encode axonemal dyneins, which power the beating of motile cilia and flagella (terms which are used interchangeably in this review). Conversely, two dyneins function as motors that transport cargo: cytoplasmic dynein-1 and -2 (Figure 1). These two dynein transport machines play critical roles in mammalian cells and many other eukaryotes, with each motor fulfilling a distinct niche.

**Box 1.**
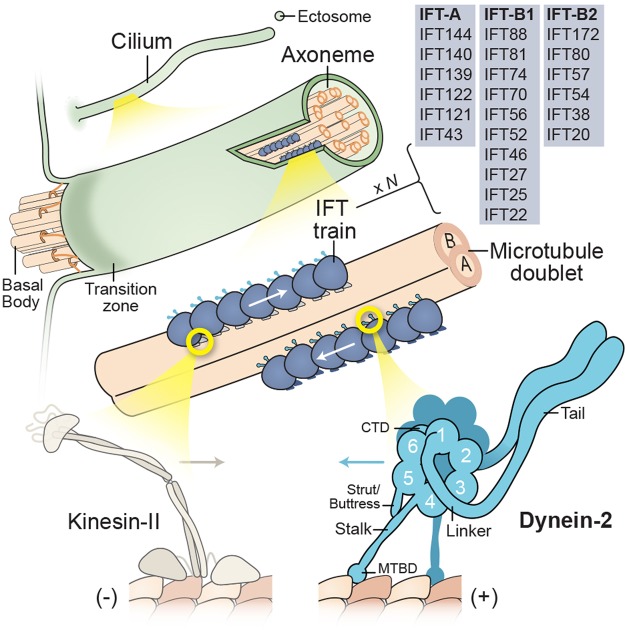
Cilia and intraflagellar transport. Cilia fall into two broad classes: motile and non-motile. Motile cilia beat with a wave-like motion to either propel cells, such as sperm and protozoa, or generate flow over the cell surface. Conversely, a non-motile primary cilium is present on almost every cell type in the human body. A widespread view was that primary cilia represented nonfunctional vestigial structures. However, landmark discoveries recast primary cilia as the ‘signaling antenna’ of the cell [149–151]. For example, mutations causing polycystic kidney disease were linked to an IFT subunit (IFT88) and a shortened cilia phenotype, supporting a sensory role for kidney primary cilia [152]. Hedgehog signaling, an important pathway for embryonic patterning, was found to depend on IFT proteins in mice [153] and involve dynamic localization of the receptor Smoothened to and from the ciliary membrane [154]. Other signaling components, involved in processes as diverse as sight, smell, taste, and appetite control, localize within cilia [150]. Moreover, receptor-containing ectosomes have recently been found to be secreted from the tip of the cilium to modulate signaling [155–158]. The core of all cilia is the axoneme, a cylindrical array of typically nine microtubule doublets that extends from the basal body. Motile axonemes normally also have a central pair of microtubules, as well as periodic arrays of axonemal dyneins and regulatory complexes that co-ordinate ciliary beating [159]. The axoneme is covered by a ciliary membrane that is continuous with the plasma membrane but distinct in protein and lipid content. During ciliary growth and maintenance, new subunits are incorporated at the ciliary tip, where the microtubule plus ends (+) reside. A diffusion barrier separates the ciliary volume and the cytoplasm [160,161]. This selective barrier involves the ‘transition zone’, a region immediately distal to the basal body characterized by Y-shaped links between the doublets and the ciliary membrane. Anterograde IFT, powered by kinesin-II motors, moves cargoes synthesized in the cytoplasm through the transition zone and toward the tip of the cilium. Conversely, dynein-2 returns cargoes to the cell body. Both motors associate with IFT trains, polymeric arrays involving two sub-complexes, IFT-A and IFT-B (consisting of IFT-B1 and IFT-B2) [162,163]. Genetically, IFT-B proteins tend to be critical for anterograde IFT and ciliogenesis, while IFT-A proteins are typically linked with retrograde IFT. However, the functions of IFT-A and -B are not so simply separated, as IFT-B proteins can be involved in cargo export, while IFT-A proteins are required for ciliary entry of a subset of membrane proteins via the adaptor protein TULP3 [164]. Structurally, dynein-2 comprises a tail domain and a motor domain containing the linker, a ring of six AAA+ modules (1–6), a coiled-coil stalk with the MTBD at its tip, a shorter coiled-coil strut/buttress, and a C-terminal domain (CTD).

Cytoplasmic dynein-1 (referred to here as ‘dynein-1’) was first isolated as a high-molecular weight ATPase (adenosine 5′-triphosphatase) with biochemical, structural, and motile properties distinct from those of kinesin; the motor driving movement to microtubule plus ends [5,6]. Since then, it has emerged that dynein-1 powers the minus-end-directed movement of a wide range of intracellular components. These cargoes span membranous organelles, nucleic acids, viruses, misfolded proteins, and cytoskeletal components [7,8]. To perform its functions, dynein-1 interacts with a battery of regulatory partners including dynactin, Lis1, NudE/NUDEL, and adaptors that link the dynein–dynactin complex to cargo [9–11]. Strikingly, mutation of these components can cause neurodevelopmental or neurodegenerative disorders [12], underscoring that the elongated cells of the nervous system are particularly sensitive to defects in dynein-1 and microtubule-based transport.

In contrast with dynein-1's discovery through protein biochemistry, the first evidence for dynein-2 (also known as dynein-1b or intraflagellar transport dynein) came from cDNA analysis. Gibbons et al. [13] identified a transcript in sea urchin embryos that is up-regulated during regeneration of cilia, but whose sequence is more similar to dynein-1 than to axonemal isoforms. This led to the suggestion that the transcript may encode a novel cargo-transporting dynein that participates in ciliary construction [13,14]. An alternative proposal held that dynein-2 functions in Golgi organization [15]. Support for the role of dynein-2 in cilia came from studies of the green alga *Chlamydomonas*. Using differential interference contrast microscopy, Rosenbaum and colleagues had discovered intraflagellar transport (IFT): a bidirectional transport system that moves material along the length of cilia [16] and is critical for their construction and maintenance [17]. Analyses of *Chlamydomonas* and *Caenorhabditis elegans* mutants lacking putative dynein-2 subunits revealed short cilia with bulbous accumulations at their tips [18–21], as though retrograde IFT of cargoes from the tip to the base of the cilium was defective.

Based on studies in a range of organisms, dynein-2 is now recognized as the ubiquitous motor for retrograde IFT in motile and sensory cilia (Box 1) [22]. The IFT system moves axonemal building blocks, regulatory proteins, and signaling molecules within the cilium [23–26] and powers surface gliding locomotion of protozoa [27,28]. It involves the interplay of dynein-2 with plus-end-directed kinesin-II motors and ∼22 IFT proteins [29–32]. These components co-assemble into polymeric IFT ‘trains’ [32–34] that move to and from the ciliary tip and bind cargoes, either directly [35] or via associating factors such as the BBSome [36] and Tulp3 [37]. Mutations in dynein-2 are associated with a group of ‘ciliopathies’ encompassing Jeune Syndrome, short rib polydactyly, and asphyxiating thoracic dystrophy [38].

The existence of dynein-1 and -2, two cargo-transporting dyneins with distinct biological functions, raises questions about which aspects of their mechanisms are conserved and which have diverged. The premise of this review is to compare dynein-1 and -2 in terms of their operating environment, subunit composition, motility, and regulation, asking: which molecular adaptations may engender their physiological roles? For comprehensive reviews of each dynein individually, the reader is referred to recent articles [7–11,22].

### Operating context

#### Dynein-1

Dynein-1 has varied modes of action in intracellular organization, mitosis, and cell migration. Among these, the transport of vesicles using coiled-coil cargo-adaptor proteins is perhaps the best understood. Here, dynein-1 assembles with its ubiquitous cofactor dynactin and a cargo adaptor, such as BicD2. The adaptor coiled coil lies in-between dynein-1 and dynactin, stabilizing their binding, while its distal end binds to a receptor specific to the cargo (Figure 1) [39]. Assembly of this complex activates dynein-1 motility [40,41]. Based on the predicted length of coiled coil within activating adaptors, dynein-1 may lie over 50 nm from the receptor on the cargo surface, although detailed images of dynein-1 in the act of cargo transport are lacking.

Cargo adaptors can recruit either one or two dynein-1s per dynactin, with two dyneins conferring transport with elevated speed and force [42,43]; parameters that are also tuned by the Lis1/NudE system [10,11]. Moreover, several dynein–dynactin units may cluster together on the vesicle surface, facilitated by lipid microdomains [44]. Absolute quantification of the dynein-1 copy number per cargo is challenging but is estimated at ∼1–5 for neuronal vesicles [45]. Collective force production by dynein-1 is thought to enable cargo transport through the crowded, viscous cytoplasm at the rapid speeds (typically 0.5–2 µm s^−1^) observed in living cells.

Many dynein-1 cargoes recruit members of the kinesin-1, -2, or -3 family, and switch between bouts of plus- and minus-end-directed movement. The directional balance can be controlled at multiple levels, including motor composition, adaptor phosphorylation, and microtubule-associated proteins (MAPs) [46]. The microtubule itself is a further important variable in dynein-1's operating environment. Surface-exposed C-terminal tails of α- and β-tubulin are subject to diverse modifications, including removal and re-addition of α-tubulin's C-terminal tyrosine [47,48]. A CAP-Gly domain within dynactin recognizes tyrosinated microtubules, making dynein-1 transport events more frequent on this subset of tracks [49–51]. These represent just some of the factors that impinge on dynein-1 cargo transport, a rapidly advancing area that has been reviewed recently [7,9–11].

#### Dynein-2

Dynein-2 transport displays pronounced differences compared with dynein-1. First, rather than involving small groups of motors bound to cargo via dynactin, dynein-2 transport occurs in the context of long linear IFT trains [33,34], which can contain dozens of motors [52] (Figure 1). Second, instead of stochastic bouts of back-and-forth motion typical of dynein-1 transport, dynein-2 operates in a highly co-ordinated fashion with the anterograde IFT motor, kinesin-II [53]. Dynein-2 is carried as a passenger on anterograde IFT trains to the ciliary tip, and then, following IFT train remodeling, powers return transport to the ciliary base. Thus, dynein-2 has strongly preferred ‘turnaround zones’ at the ciliary tip and base, in which it interconverts between inactive and active states. Third, rather than involving conventional microtubule tracks, dynein-2 moves on microtubule doublets of the axoneme, with each doublet having an unusual structure consisting of one complete ‘A-tubule’ fused to an incomplete ‘B-tubule’ (Box 1), and each tubule bearing different MAPs and post-translational modifications [48,54]. Interestingly, it was recently discovered that in *Chlamydomonas reinhardtii*, retrograde IFT occurs on the A-tubule and anterograde IFT occurs on the B-tubule, suggesting a means to prevent collisions between oppositely directed IFT trains [34]. Fourth, dynein-2 operates in the highly confined space between the doublet outer surface and the ciliary membrane [32]. Much of this space is occupied by the IFT train itself, which is closely appressed to the lipid bilayer [33]. Thus, dynein-2 is likely to experience the IFT train and underlying microtubule doublet with extremely high effective concentration. Moreover, dynein-2 probably experiences further confinement as it exports cargoes from the cilium through the ‘transition zone’; part of the diffusion barrier that separates the ciliary volume from the cytosol (Box 1).

### Subunits

**Figure 2.**
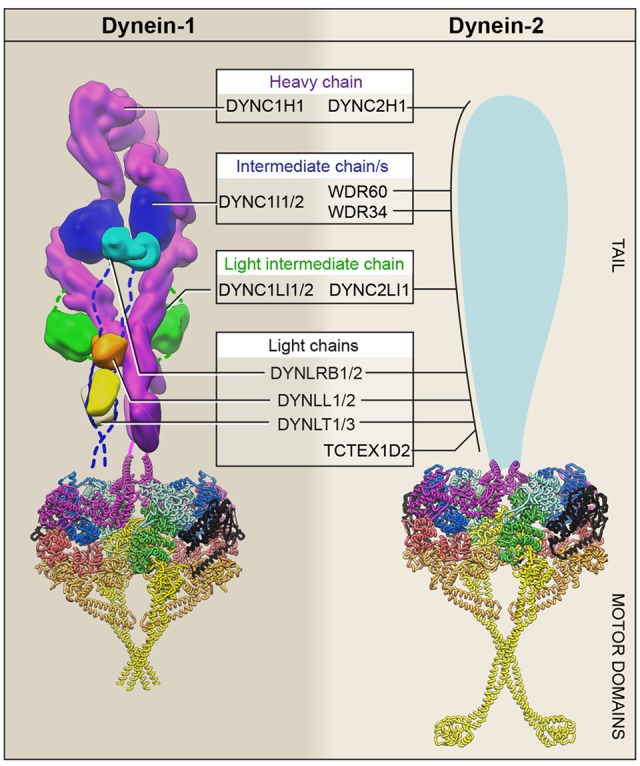
Dynein-1 and -2 subunit composition. Summary of unique and shared components in dynein-1 and -2. The C-terminal region of each heavy chain forms the motor domain, while the N-terminal region forms the tail and associates with intermediate, light-intermediate, and light chains. In mammals, there are two isoforms for each class of dynein-1-associated subunit. For example, the two intermediate chain isoforms as denoted here as ‘DYNC1I1/2’. Structural information is available for the dynein-1 and -2 motor domains, which are shown in their auto-inhibited ‘phi-particle’ state in ribbon representation (colored as in Figure 3) [57,92,126]. The dynein-1 tail is shown in surface representation [57]. The unknown architecture of the dynein-2 tail is shown schematically.

Dynein-1 and -2 both function as multi-protein complexes built around a pair of force-generating heavy chains (Figure 2). In mammals, the dynein-1 heavy chain is DYNC1H1 and the dynein-2 heavy chain is DYNC2H1 (see [55] for a useful table of nomenclature in other organisms). Each ∼0.5 MDa heavy chain consists of an N-terminal tail (∼1300 amino acids) attached to a C-terminal motor domain (∼3300 amino acids). In dynein-1, the tail mediates dimerization and interactions with associated subunits, regulatory proteins, and cargoes. The tails of human dynein-1 and -2 share relatively low sequence identity (14%) compared with the motor domains (29%), indicating that the tail may have diverged to facilitate different functions and/or regulatory programs.

#### Dynein-1

The dynein-1 heavy chain associates with five further types of subunit: the intermediate chain (DYNC1I), light-intermediate chain (DYNC1LI), and three classes of light chain (DYNLRB, DYNLL, and DYNLT; also known as Roadblock, LC8, and TCTEX). Like the heavy chains, each subunit is present as a dimer. In mammalian genomes, there are two isoforms for each class of associated subunit, whose products are further diversified by alternative splicing and phosphorylation. The full functional consequences of these different isoforms are still emerging; progress toward this goal is reviewed in ref. [56]. The dominant assembly state of the dynein-1 subunits appears to be homodimeric, with different isoforms giving rise to complexes with specialized properties in mammalian tissues [56].

Insights into dynein-1 subunit architecture are available from recent high- and intermediate-resolution electron microscopy (EM) structures [42,43,57–59], building on earlier biochemical, X-ray, and NMR studies (Figure 2). The tail of the heavy chain forms a rod-like series of α-helical bundles [57], which dimerize via a compact N-terminal domain [59]. The intermediate chains bind directly to the heavy chain using a C-terminal seven-bladed β-propeller domain [42]. Intermediate chain dimerization is mediated by extended N-terminal regions, which are held together by the three classes of light chain [60,61]. Finally, the light-intermediate chain, whose core is a globular Ras-like domain [62], associates tightly with the heavy chain, inserting flanking N- and C-terminal α-helices within its fold [42]. Overall, the 12 polypeptides of the dynein-1 tail generate an extended structure, with a surface area of ∼150 000 Å^2^ in principle available for protein–protein interactions.

What are the functions of the associated subunits? Many of the dynein-1-associated subunits have been implicated in direct or indirect cargo binding [7]. For example, the light-intermediate chain C-terminal region interacts with a variety of cargo adaptors, helping to stabilize their association with the dynein-1/dynactin complex [62–64]. Moreover, the N-terminal α-helix of the intermediate chain binds to the dynein-1 regulators dynactin p150 and NudE/Nudel [65,66]. The associated subunits also play fundamental architectural roles in dynein-1. For example, the heavy chains are constrained in a side-by-side arrangement by binding of the intermediate and light chains [59]. In order for the dynein-1 tail to bind dynactin and cargo adaptors, one heavy chain must undergo a major rotation relative to the other, bringing the two heavy chains close to parallel [57]. The intermediate chains stabilize this parallel configuration, with the β-propeller on one heavy chain interacting in *trans* with the adjacent heavy chain [42]. When two dyneins are bound per dynactin, a network of intermediate chain:heavy chain contacts is formed [42]. These interactions are likely to rigidify the dynein–dynactin complex and help orient the attached motor domains for movement on the microtubule.

#### Dynein-2

The subunit composition of dynein-2 has been slower to emerge than that of dynein-1. Dynein-2 has not yet been purified to homogeneity from native sources, owing in part to its comparative lability and scarcity. To date, its components have been characterized by pioneering genetic, cellular, and biochemical experiments in a variety of systems. These studies indicate that, as well as the heavy chain (DYNC2H1), dynein-2 contains the following components that are related but distinct from their counterparts in dynein-1: a light-intermediate chain (DYNC2LI1) [67–71], a light chain (TCTEX1D2) [72–74], and notably two types of intermediate chain (WDR34 and WDR60) [72,75–81]. Splice variants of the light-intermediate chain have been reported [82]. Proteomic studies suggest that human dynein-2 additionally associates with all of the Roadblock, LC8, and TCTEX isoforms found in dynein-1 [72]. A current view of dynein-2 subunit composition is summarized in Figure 2. For dynein-1, it has been reasonable to suppose that its subunit complexity reflects the large number of cargoes that it transports. It is thus somewhat unexpected that dynein-2, which is thought to interact with cargoes principally via the IFT train and BBSome, contains such a variety of subunits, raising questions about their function.

How do the architecture and stoichiometry of subunits in dynein-2 compare with dynein-1? The structure of the dynein-2 tail is unknown. Sedimentation analysis and EM suggest that the dynein-2 heavy chain forms a dimer akin to dynein-1 [15,71,83]. In *C. reinhardtii*, a putative monomer fraction has also been detected [71,75]. The light-intermediate chain associates with the heavy chain in either context. A major point of departure from dynein-1 appears to be the two intermediate chains. Termed WDR34 and WDR60 in mammals, these subunits were discovered as FAP133 and FAP163 in *C. reinhardtii* [75,80]. Both contain a predicted C-terminal β-propeller, attached to a highly divergent N-terminal region of ∼140 and ∼620 amino acids in WDR34 and WDR60, respectively. While neither N-terminal region contains consensus light chain-binding sites, two degenerate LC8-binding sites have been predicted in WDR34. Congruently, LC8 co-purifies with FAP133/WDR34 in *C. reinhardtii* [75]. There is compelling evidence that, in contrast with the dynein-1 intermediate chain, WDR34 does not homodimerize: the endogenous protein fails to co-immunoprecipitate with GFP (green fluorescent protein)-tagged WDR34 expressed in RPE1 cells [72]. Rather, biochemical studies point to a model in which WDR34 exists in a sub-complex with WDR60 and light chains, which in turn forms a metastable interaction with the dynein-2 heavy chain and light-intermediate chain [22,72,75,80].

Dynein-2 subunit composition varies in some organisms. For example, trypanosomatids feature two dynein-2 heavy chain genes rather than one [84]. In *Trypanosoma brucei*, both heavy chains exist in the same complex and very likely form a heterodimer [85]. Heteromeric motor subunits are found in axonemal dyneins but are rare among cargo-transporting motors. A notable precedent is the anterograde IFT motor, kinesin-II, whose major form contains a heterodimer of kinesin heavy chains (as well as an ancillary subunit, KAP, hence its common name ‘heterotrimeric kinesin-II’) [86,87]. Pairing of two different force-generating subunits has the potential to tune the motile properties of the resulting heterodimer [88–90]. It is also possible that, evolutionarily, heterodimerization served as an efficient means to generate a unique binding interface for the IFT train. In this scenario, it is conceivable that dynein-2's acquisition of two distinct intermediate chains served a similar role.

### Motor domains

**Figure 3.**
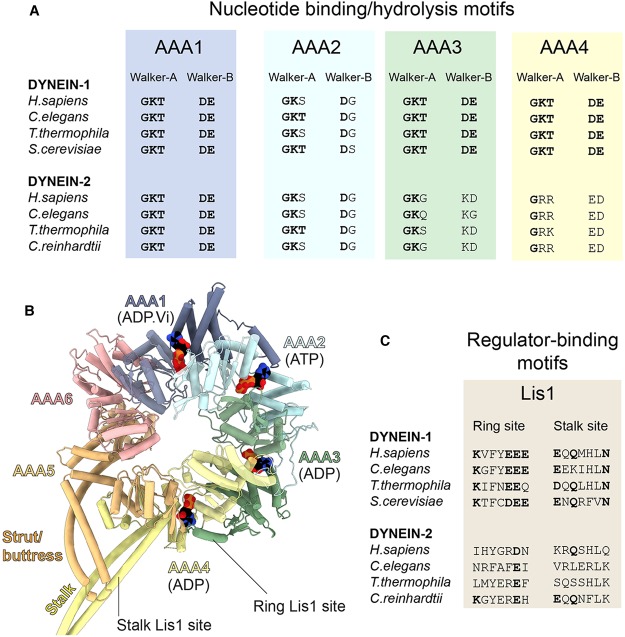
Dynein motor domain structure and motif conservation. (**A**) Analysis of the Walker-A and -B motifs within the AAA+ modules of dynein-1 and -2 from different species. Within the Walker-A consensus sequence (GKT), the lysine is important for nucleotide binding. Within the Walker-B consensus sequence (DE), the glutamic acid is thought to be the catalytic base that polarizes H_2_O for an inline attack on the γ-phosphate of ATP. Amino acids matching the consensus sequence are shown in bold type. In dynein-2, the Walker-A and -B motifs of AAA3 and AAA4 deviate from the consensus, in contrast with the situation in dynein-1. Beyond the AAA+ modules, Redwine et al. [165] have noted differences in charged amino acids in the MTBD of dynein-1 and dynein-2, which are likely to influence their respective microtubule affinities. (**B**) Structure of the dynein-2 AAA+ ring from PDB 4RH7 [92], with α-helices shown as cylinders and nucleotides in space-filling representation. The linker domain, which would lie on the near face of the AAA+ ring, and the CTD, which would lie on the far face, are omitted for clarity. (**C**) Analysis of Lis1's binding sites in the AAA+ ring and stalk. Key charged and polar amino acids (bold) shown to be important for Lis1 binding in *S. cerevisiae* dynein-1 [108,110] are not conserved in dynein-2.

Dynein-1 and -2 use a structurally similar motor domain to convert the energy from ATP hydrolysis into movement [91,92]. This C-terminal region of the heavy chain can be recombinantly expressed as an active monomer [93,94]. Its enzymatic core is a ring of six AAA+ (ATPases associated with various cellular activities) modules (AAA1–6) with an associated C-terminal domain (CTD) [95]. A rod-like mechanical element, termed the ‘linker’, arches over the AAA+ ring and amplifies conformational changes within it [96]. Protruding from the AAA+ ring are a ∼15 nm antiparallel coiled-coil ‘stalk’, tipped by a microtubule-binding domain (MTBD) [97] and a coiled-coil ‘strut/buttress’ that interacts with the stalk (Box 1 and Figure 3B) [98,99]. These structural elements are coupled, such that nucleotide transactions within the AAA+ ring influence the affinity of the MTBD and the conformation of the linker, and *vice versa*, enabling mechanochemical cycles of movement along the microtubule.

Within this similar fold, are there differences in functional motifs that could influence dynein-1 and -2 motility? Two hallmarks of the AAA+ module are the Walker-A and -B motifs, which are crucial for ATP binding and hydrolysis, respectively [100]. Across dyneins, only modules AAA1–4 possess a functional Walker-A motif and bind nucleotide. Dynein motility is driven by the ATPase activity of AAA1 [101]. Accordingly, the Walker-A and -B motifs of AAA1 strictly adhere to the consensus sequence found in other AAA+ proteins (Figure 3A). In both dynein-1 and -2, the Walker-A motif of AAA2 is well conserved but its Walker-B motif deviates from the consensus, consistent with the idea that AAA2 stably binds ATP and does not hydrolyze it [102]. In contrast, the properties of AAA3 and AAA4 may differ between dynein-1 and -2.

#### Dynein-1

In dynein-1, AAA3 and AAA4 display conserved Walker-A and -B motifs (Figure 3A). X-ray structures show that within AAA3, these motifs are well positioned to participate in ATP binding and hydrolysis, whereas in AAA4 the Walker-B glutamate is displaced [91,102]. Disabling ATP binding or hydrolysis at AAA3 impairs dynein-1's ability to release from microtubules and undergo conformational change; equivalent mutations in AAA4 have a milder effect [101,103,104]. Single-molecule studies with a slowly hydrolyzable ATP analog (ATPγS) and pre-steady state kinetics are consistent with a model in which AAA3 rapidly hydrolyzes ATP, then retains ADP bound while AAA1 undergoes multiple ATPase cycles [91,105]. Thus, AAA3 may spend most of its time in an ADP state and periodically transition through apo and ATP/ADP-Pi states associated with altered microtubule affinity [105,106]; a process that may be influenced by external load [107]. Interestingly, Lis1 — a ubiquitous dynein-1 regulator — is sensitive to the nucleotide state of AAA3. As a dimer, Lis1 binds via one of its β-propeller domains at the AAA3–AAA4 junction (Figure 3B, ‘ring site’) and promotes microtubule attachment [108,109]. When AAA3 is in an ATP state, Lis1's other β-propeller can access a second binding site in dynein's stalk (Figure 3B, ‘stalk site’), weakening the strong microtubule affinity associated with the AAA3 ATP state [110]. Thus, nucleotide transactions at AAA3 can change dynein-1 microtubule binding directly, as well as via Lis1, providing multiple opportunities for regulation [10,11,111–114].

#### Dynein-2

In dynein-2, the AAA3 and AAA4 Walker-B motif deviates from the consensus sequence, suggesting that these modules are unlikely to hydrolyze ATP [102] (Figure 3A). Moreover, neither the ring or stalk Lis1-binding sites are conserved in dynein-2 (Figure 3C), suggesting that Lis1 AAA3 nucleotide sensing is unlikely to be present in dynein-2. Insight into the nucleotide status of AAA3 and AAA4 in dynein-2 comes from a crystal structure of the motor domain as a monomer [92]. Although crystallized in the presence of ATP and vanadate, both AAA3 and AAA4 were found to contain ADP. Furthermore, the dynein-2 motor domain was observed to co-purify with three stably-bound nucleotides, likely corresponding to ATP in AAA2 and ADP in AAA3 and AAA4 [92]. These data indicate that dynein-2's AAA3 and AAA4 modules may exist in a stable ADP-bound state. If true, dynein-2 would present an appealing model for kinetic studies of dynein, as these have generally been complicated by the presence of multiple ATPase sites. It would also imply that ATP turnover at AAA3 and AAA4 is not required for the precise regulation of dynein-2 motility apparent during IFT.

### Motility and regulation

#### Dynein-1

Rapid progress has been made in understanding dynein-1 motility, catalyzed by the availability of native and recombinant purification systems, single-molecule assays, and structural information. A well-studied model system is *Saccharomyces cerevisiae* dynein-1, which is an intrinsically active motor [115]. When its two motor domains are paired (either natively via the tail or by appending a dimerizing moiety to the motor N-terminus), the resultant dimer can move continuously (‘processively’) along the microtubule for several microns before detaching, at peak velocities of ∼0.1 µm s^−1^, with a stall force of ∼4–7 pN [115,116]. Dynactin increases the intrinsic run length of *S. cerevisiae* dynein-1 by ∼2-fold [117].

Conversely, isolated mammalian dynein-1 displays a variety of behaviors on the microtubule, including non-directional diffusive motion, static binding, and short processive runs [40,41,118]. Processive movement of mammalian dynein-1 is strongly stimulated by the binding of dynactin and a cargo adaptor [40,41]. The resulting ternary complexes can move over many microns, at peak speeds of ∼0.8 µm s^−1^, with stall forces of ∼4 pN [119]; values which are further increased when two dynein-1s are present per dynactin [42]. Thus, dynactin and cargo adaptors are said to ‘activate’ the processive motility of mammalian dynein-1 [7].

The transition between non-motile and highly processive forms of mammalian dynein-1 involves a large structural change. Early EM images of dynein-1 showed that a subset of molecules adopt a compact conformation, in which the two motor domains are closely apposed [120]. These molecules were named ‘phi-particles’ because, with their tails and stalks protruding from opposite ends of the motor domains, they had a morphological resemblance to the Greek letter ϕ (Figure 2). Torisawa et al. [121] proposed that the phi-particle corresponds to a non-motile, auto-inhibited state of dynein-1. Analysis of the phi-particle by cryo-EM revealed that the motor domains stack against each other in a rotationally symmetric (C2) manner, trapping them in a conformation with weak affinity for microtubules [57]. The tails also have near 2-fold rotational symmetry. However, when bound to dynactin and a cargo adaptor, the heavy chains adopt an approximately parallel arrangement, similar to the translational symmetry of dynactin's actin-related filament [42,43,57–59,122]. These data indicate that binding of dynein-1 to dynactin and a cargo-adaptor is coupled to the opening of the auto-inhibited form of the dynein-1 motor domains and their rearrangement into a parallel configuration that is optimal for processive movement on the microtubule [57]. Several aspects of this important transition remain to be elucidated, including its kinetics, regulation by multiple motors, and interplay with dynactin binding to the microtubule and intermediate chain N-terminus. It is also unclear to what extent dynactin and cargo adaptors force apart the phi-particle or capture a transiently open form.

#### Dynein-2

To date, many of the insights into dynein-2 motility have come via observation of retrograde IFT in living cells. These studies reveal that the velocity of retrograde IFT varies widely among species, from ∼0.4 μm s^−1^ (mouse IMCD3 cells) to ∼3 μm s^−1^ (*C. reinhardtii*) to ∼7 μm s^−1^ (*T. brucei*) [123], and can even differ within segments of the same cilium [124]. Retrograde IFT also generates large forces. Optical trapping of beads attached to IFT-engaged membrane proteins shows that retrograde trains are able to move against resisting loads of over 25 pN [27,125]. Production of large forces, which may be important for translocating cargoes through the transition zone or densely packed ciliary membrane, is consistent with the collective action of multiple dynein-2 motors per retrograde train [27,125].

Recently, these studies of retrograde IFT in living cells have been complemented by analysis of dynein-2 motility *in vitro* [83,92,126,127]. Monomeric human dynein-2 motor domains are capable of powering fast (∼0.5 µm s^−1^) movement in ensemble microtubule-gliding assays, comparable to the rate of retrograde IFT in mammalian cells [126]. Strikingly, the activities of the motor domain are inhibited upon dimerization. EM and mutagenesis showed that the basis of this auto-inhibitory effect is stacking of the motor domains against one another, in a manner incompatible with dynein motility in at least three ways [126]. First, the linker domains are trapped at the motor–motor interface, preventing them from amplifying conformational change; Second, the MTBDs point in opposite directions, meaning that they cannot simultaneously attach to the microtubule. Third, the stalks interact as they cross one another, likely restricting conformational changes required for communication between the AAA+ ring and MTBD (Figure 2). Contemporaneous studies revealed that the auto-inhibitory architecture in dynein-2 is essentially identical to the rotationally symmetric arrangement of dynein-1 motor domains found within the phi-particle [57,126]. These studies show that mammalian dynein-1 and -2 share a common mode of auto-inhibition. Stated differently, they raise the idea that the phi-particle is an ancient control mechanism that was present in the last common ancestor of transport dyneins.

The finding that the dynein-2 motor domains exist in an auto-inhibited state suggested a model for dynein-2 regulation during IFT [126]: following dimerization in the cytoplasm, dynein-2 adopts its switched-off conformation, enabling the motor to be loaded onto anterograde IFT trains at the ciliary base and transported tip-ward by kinesin-II with minimal resistance. Consistent with this notion, auto-inhibition of dynein-2 facilitates its anterograde transport by kinesin-II *in vitro* [126]. At the ciliary tip, the dynein-2 motor domains must be activated via disruption of the auto-inhibitory interface, releasing the linker and stalk domains for motility. Thus, a question posed by this model is how dynein-2 is unstacked and activated at the ciliary tip.

Classic dynein-1 regulators, such as dynactin, would seem plausible candidates to influence dynein-2's auto-inhibited conformation. However, organisms such as *Chlamydomonas* lack dynactin subunits in their genome yet display canonical retrograde IFT. Furthermore, in mammalian systems, dynactin does not co-immunoprecipitate with dynein-2 [72] and is not found within cilia [128]. These observations argue against the involvement of dynactin in dynein-2 regulation, indicating that the mechanism of dynein-2 activation may be unique.

There is mixed evidence for Lis1 in cilia. In *C. reinhardtii*, a direct Lis1 ortholog is absent (although a Lis1-related protein has been identified [129] and found to associate with axonemal outer-arm dynein, probably as a monomer [130]). In *C. elegans*, Lis1 has been observed in the middle segment of phasmid cilia, where it undergoes occasional IFT-like movements [131]. In mammals, Lis1 has been reported in the primary cilia of RPE1 cells, but it does not co-immunoprecipitate with dynein-2 [72]. Conversely, Lis1 is absent in the primary cilia of NIH3T3 cells [129]. Moreover, because Lis1's binding sites in the AAA+ ring and stalk are not conserved in dynein-2, it is unlikely that Lis1 could interact with dynein-2 via these surfaces (Figure 3C). In summary, while the role of Lis1 in the subset of primary cilia in which it is found is mysterious, evidence suggests that Lis1 is not a core component of the dynein-2 machinery in contrast with its essential roles in dynein-1 function.

Among the best candidates to regulate dynein-2 are subunits of the IFT train itself [72]. These ∼22 proteins fall into the IFT-A and IFT-B complexes, which form oligomeric arrays of several hundred nanometers within cilia (Box 1) [31]. Dynein-2 does not co-purify with IFT proteins in a stoichiometric fashion, indicating that any interactions between them may be weak and/or coupled to IFT train oligomerization.

In the simplest scenario, two modes of interaction between dynein-2 and the IFT train can be envisaged: one involving auto-inhibited dynein-2 that is used during anterograde transport of the motor to the ciliary tip, and another, involving active dynein-2, evoked during retrograde transport. Through molecular genetic studies, the largest protein in the IFT-B complex, IFT172, has been implicated in the anterograde transport of dynein-2 and/or IFT train turnaround at the ciliary tip [132–134]. IFT172 is thus a candidate to interact with dynein-2 in its auto-inhibited conformation. Conversely, the IFT-A complex appears to be important for retrograde transport, making it a candidate to participate in dynein-2 activation (Box 1). Dysfunction of IFT-A is typically associated with short cilia with accumulations at their tips, similar to the cellular phenotype of dynein-2 mutants [31]. The IFT-A proteins IFT139 and IFT140 have been found to co-immmunoprecipitate with dynein-2 in *C. reinhardtii* [75] and mammalian cells [135], respectively. In general, because the IFT-A and IFT-B proteins play multiple roles in IFT train assembly, cargo binding, and ciliogenesis, dissecting their specific contribution to dynein-2 regulation is an ongoing challenge.

Sequence analysis suggests that IFT-A proteins share an evolutionary ancestry with the membrane coat proteins COPI, COPII, and clathrin [136]. Thus, IFT-A is likely to have more in common with these ‘coatomer’ complexes than with the dynein-1 cofactor, dynactin. The IFT-B protein IFT172 also contains WD40 and TPR repeats characteristic of coat proteins [137]. In dynein-1, long (>200 residue) coiled-coil domains are a signature of activating adaptors that stabilize the interaction between the dynein-1 tail and dynactin [7]. While long coiled-coil regions are predicted in the IFT-B proteins IFT-81 and -74 [137], it is unknown whether they interact with dynein-2 or if the use of coiled-coil adaptors is specific to the dynein-1 machinery.

Anterograde IFT trains are ‘remodeled’ at the ciliary tip [138]. This process involves cargo release and IFT subunit disassembly, mixing, and reassembly into retrograde trains — all within 1–2 s [25,139,140]. When viewed along their long axis, anterograde trains have a double-row appearance, whereas retrograde trains are narrower, suggesting that they are differentiated by a significant architectural change [34]. It is possible that remodeling of the IFT subunits uncovers an activating binding site for dynein-2 that re-orients its motor domains into a parallel configuration for retrograde motility [126]. A non-mutually exclusive idea is that dynein-2 is regulated by local post-translational modification at the ciliary tip. For instance, the anterograde motor kinesin-II is proposed to be regulated by a tip-localized kinase [141,142]. Reduction in cAMP-dependent protein kinase activity is important for recruitment of the BBSome to the ciliary tip via Kif7 [36]; another example of the type of tip-localized biochemistry that could analogously act on dynein-2 or IFT subunits. Numerous additional questions relating to dynein-2 regulation exist. In *C. reinhardtii*, dynein-2-driven IFT trains move on the A-tubule of the microtubule doublet and not the B-tubule [34]. Immunogold EM indicates that A-tubules are enriched in tyrosinated tubulin, whereas B-tubules are detyrosinated and glutamylated [143]. Do these distinctions form part of a ‘tubulin code’ [47] that preferentially positions dynein-2 on the A-tubule [34]? It has alternatively been proposed that dynein-2 could intrinsically veer in the A-tubule direction by favoring a spiral path over linear motion [144]. In most cilia, the axoneme terminates in a tapered fashion, with the A-tubules continuing beyond the B-tubules. Does this geometry regulate IFT train turnaround and the initiation of retrograde transport [132]? It will also be important to elucidate if the ciliary membrane influences dynein-2 motility, beyond keeping the IFT subunits and microtubule doublets in close proximity and at high effective concentration. Finally, at the systems level, how is the quantity of dynein-2 entering and exiting the cilium controlled to support ciliary growth, maintenance, and signaling [145,146]?

### Outlook

As the two cargo transporters of the dynein family, dynein-1 and -2 share a number of striking similarities. These include a grossly similar dimeric architecture and a shared mode of auto-inhibition. Yet fundamental aspects of their mechanisms have diverged, such as the means by which they are activated and co-ordinate bidirectional movement with kinesins. It is tempting to speculate that some of the distinguishing features of dynein-2 and IFT, including the use of linear arrays of motors, turnaround zones at the ciliary termini, and segregation of anterograde and retrograde transport to different tracks, reflect adaptations to the cramped environment of the cilium (Figure 1). The confined nature of the cilium may also be fundamental to its roles in signaling [147]. As the differences between dynein-1 and -2 come into focus, we can anticipate a deeper understanding of each dynein individually and, perhaps, insight into the adaptive logic of cellular transport systems more broadly.

## Abbreviations

AAA+: ATPases associated with various cellular activities
ATP: adenosine 5′-triphosphate
CTD: C-terminal domain
EM: electron microscopy
IFT: intraflagellar transport
MAP: microtubule-associated protein
MTBD: microtubule-binding domain
